# COVID-19 testing in Delaware’s underserved communities: demographic and social determinants of testing inequality

**DOI:** 10.1007/s44155-022-00013-1

**Published:** 2022-06-27

**Authors:** Sharron Xuanren Wang, Nicole Bell Rogers, Melissa Harrington, Dorothy Dillard

**Affiliations:** 1 Department of Sociology and Criminal Justice, Delaware State University, Dover, DE USA; 2 Department of Nursing, Delaware State University, Dover, DE USA; 3 Department of Biology, Delaware State University, Dover, DE USA; 4 Center for Neighborhood Revitalization and Research, Delaware State University, Dover, DE USA

**Keywords:** COVID-19, Health disparities, COVID-19 testing, Underserved communities

## Abstract

**Background:**

Health experts believe that frequent COVID-19 testing is one of the most important practices for stopping the spread of the COVID-19 virus. Demographic and social factors might play a role in whether a person gets tested for COVID. This present study aimed to investigate (1) the demographic and social factors affecting a person’s likelihood of getting tested for COVID-19, and (2) the demographic and social factors related to a positive serology test (i.e., indicating likely past infection).

**Methods:**

Data were extracted from a survey conducted in Delaware’s underserved communities. Participants were asked to complete a questionnaire about their COVID-19 testing history, and nurses at the study site collected a serology sample from each participant.

**Results:**

Our results indicated that Black or Hispanic individuals living in underserved communities had greater odds of having been tested previously for COVID compared to being non-Hispanic White. In addition, our study found that being female, educated, feeling safe in one’s neighborhood, being vaccinated against COVID, and being an essential worker increased one’s odds of having been previously tested for COVID-19. Regarding the results of the COVID-19 antibody serology tests, our findings revealed that Hispanic respondents were more likely to have a positive serology test compared to non-Hispanic White respondents, indicating that the Hispanic individuals were more likely to contract the virus. Educated individuals were less likely to have a positive serology test compared to the less-educated. Those who expressed hesitancy about getting vaccinated for COVID-19 and identified themselves as essential workers were more likely to have a positive serology test and to have previously contracted the virus.

**Conclusions:**

Identifying key factors associated with COVID-19 testing may help establish novel strategies to increase testing rates among vulnerable population. Public health and policy implications are discussed in the article.

Health experts believe that frequent COVID-19 testing is one of the most important practices for stopping the spread of the COVID-19 virus. Testing is important for identifying, tracing, and treating COVID-19 cases, while also helping to inform critical policy decisions, such as whether to reopen businesses or restrict public gatherings [1]. The two main COVID-19 testing mechanisms include a diagnostic test (i.e., viral test) to detect the presence of the virus and an antibody test to detect past infection. The U.S. Food and Drug Administration (FDA) issued an Emergency Use Authorization (EUA) for the COVID-19 diagnostic test on February 3, 2020, and COVID-19 tests have been available in the U.S. since March 12, 2020. Diagnostic test results are used in the reporting of COVID-19 cases by the U.S. government and other organizations. Initially, the Centers for Disease Control (CDC) suggested that anyone having COVID-19 symptoms or anyone who had been in contact with someone diagnosed with COVID-19 should get tested. Later, following the advent of COVID-19 vaccines, the CDC suggested that unvaccinated people who participated in activities that put them at greater risk for contracting COVID-19 should also get tested.

Following the diagnostic test for COVID-19, the FDA authorized serology antibody tests for COVID-19 on Nov 6, 2020. An antibody test can detect the presence of IgG and IgM antibodies typically found in individuals with previous coronavirus infection (SARS-Co-V-2). The IgG and IgM antibodies are produced as part of the body’s immune response in both symptomatic and asymptomatic cases and can be detected as early as 14 to 48 days post-infection [2, 3]. The serology test has public health value for monitoring and responding to the ever-changing pandemic, especially when tracking past infections, which are not always detectable by tests for the virus [4]; however, both the CDC and the FDA have indicated that antibody testing cannot be used to replace tests for the virus in detecting current COVID-19 infection.

The number of infections per 100,000 persons in a population is often used in calculating the rate of COVID-19 infection for a given area. However, the reported rate of infection in the population depends on the frequency of testing in each segment of the population, as well as the fraction of positive tests among those tested. Relying on these two factors alone might be problematic for a number of reasons, for instance COVID-19 testing is not random [5]. Many factors determine whether a person gets tested. Those factors include, for example, test kit distributions in the area, whether a person knows where to get tested, taboos associated with a positive COVID test, whether the person is experiencing COVID symptoms and is familiar with the symptoms of COVID, etc.

Demographic and social factors might also play a role in whether a person gets tested for COVID [6]. For instance, COVID exposure might be associated with occupation. Indeed, essential workers have a higher risk of exposure to the virus. Moreover, people living in underserved communities across the U.S. are less likely to get tested for COVID, but more likely to receive a positive result when tested [5]. Racial and ethnic health disparities in the U.S. have also been reinforced by the COVID-19 pandemic. African Americans and Latin/Hispanic Americans face a disproportionately negative impact of the pandemic and are more likely to experience negative health outcomes related to COVID-19. Studies have suggested that being Black or Hispanic is associated with increased risk of COVID-19 infection and greater disease severity, and these effects may be attributed to socioeconomic factors [7]. For example, Black and Hispanic individuals are more likely to be essential workers and have lower access to healthcare [8]. Because of the rapid transmission rate of COVID-19, those who work in high-risk settings such as assembly line and food processing plants, or in public or customer service are at far greater risk have accounted for more than 50% of positive case in some areas of the United States [8]. Statistical data also indicates that 1 in 555 Black Americans have died from COVID-19 since the start of the pandemic. While Black Americans are at increased risk for exposure and infection, men across all racial and ethnic backgrounds are more likely to succumb to COVID and COVID-related deaths, when compared to females [8–11].

Disadvantaged populations, including racial minorities and people living in underserved communities, might have limited access to COVID tests or might not understand the importance of getting tested. Therefore, examining only the positive rates in existing datasets might be misleading due to under-testing of some populations [5, 12]. The goal of this present study was to explore the effects of demographic, social, and pandemic-related characteristics on COVID-19 testing. Importantly, this study gathered not only self-report data about an individual’s COVID-19 testing history and past test results, but also included serology testing on participants to detect recent past infection from the presence of COVID-19 antibodies. The serology test is important because people who never presented symptoms of COVID infection might never have been tested, whereas the serology test could detect past infection regardless of symptoms. Therefore, analyzing results from both the serology tests and diagnostic test histories may help to provide a more comprehensive picture of COVID-19 testing and infection in underserved communities. Specifically, this study takes place in the state of Delaware and aims to investigate (1) the demographic and social factors affecting a person’s likelihood of getting tested for COVID-19, and (2) the demographic and social factors related to a positive serology test (i.e., indicating likely past infection). Thus, the current study hopes to contribute to the literature of understanding the factors influencing COVID-19 testing and the social determinants of testing inequality in underserved communities.

## Methods

### Recruitment

Participants were recruited from the most underserved communities across Delaware’s three counties (New Castle County, Kent County, and Sussex County). Nine communities (three from each county) were selected based on the Community Health Index (CHI) scores. Delaware’s Division of Public Health (DE DPH) uses the Community Health Index (CHI) as a common indicator for characterizing community health at the census tract level. The CHI is a composite score derived from several measures including life expectancy, infant mortality rate, percent of high school graduates, and child poverty rates [13]. The communities where we conducted the present study ranked low in terms of these community health measures. Moreover, as expected, these communities have concentrated poverty, limited health resources, and large minority populations. For recruitment in these communities, we collaborated with two trusted community health advocacy agencies: the Wilmington Hope Commission (WHC) and the Sussex County Health Coalition (SCHC). The WHC and SCHC currently serve as two of the key coordinating organizations in carrying out Delaware’s COVID-19 response. They also have well-established networks in the communities of interest, including relationships with community-based healthcare providers and clinics, non-profit organizations, and faith-based organizations. The community-health providers and clinics have earned a great deal of trust and a strong rapport among the residents in the communities. We worked with the WHC and SCHS to (1) develop a recruitment protocol, recruitment materials, and information appropriate for the residents of the study sites; (2) establish partnerships with the community-based organizations and clinics serving the residents; and (3) maintain working relationships with the study sites.

Before data collection began, graduate nursing students and community/public health nurses were recruited and trained to conduct serology testing. The graduate students were currently enrolled in the online MSN (Masters of Science in Nursing) program. Through community partnerships with local hospitals and other health care organizations, additional nursing support was acquired to assist with data collection. The training process involved addressing study protocols, human subjects training, discussing nursing roles and conduct. Serology testing procedures, and infection control practices were also reviewed. The nurses were trained on proper data collection and infection control protocols aligned with those of Centers for Disease Control (CDC) and the World Health Organization (WHO).

### Data and method

Antibody data is collected using rapid serology testing with COVID-19 lateral flow serology test kits (ASSURE^®^; MP Biomedicals, Solon, OH). Participants were assessed by a nurse using a COVID-19 screening tool adopted from the CDC (2021) to ensure that the participant did not have symptoms of active COVID-19 infection. Once the screening tool was completed, informed consent was obtained. For participants with reported symptoms, study participation was deferred until symptoms and infection had resolved.

Antibody serology testing were collected via use of lateral flow immunoassays. Small capillary blood (10 μl) specimens (fingerstick) were obtained via a single-use lancet and were added to the lateral test cassette sample well. These devices can detect the presence of IgG and IgM antibodies that are typically found in those individuals who have experienced previous coronavirus infection (SARS-Co-V-2.) Typically, these antibodies (i.e., IgG and IgM) are produced as part of the body’s immune response. These antibodies have been found to be present in those who were symptomatic or asymptomatic as early as 14 days after symptom onset [2, 3]. The sensitivity and specificity of the test were found to be greater than 90% for detecting the presence of viral infection from samples of human blood [3], with IgG antibodies detectable more than four months post-infection [13].

**Fig. 1:**
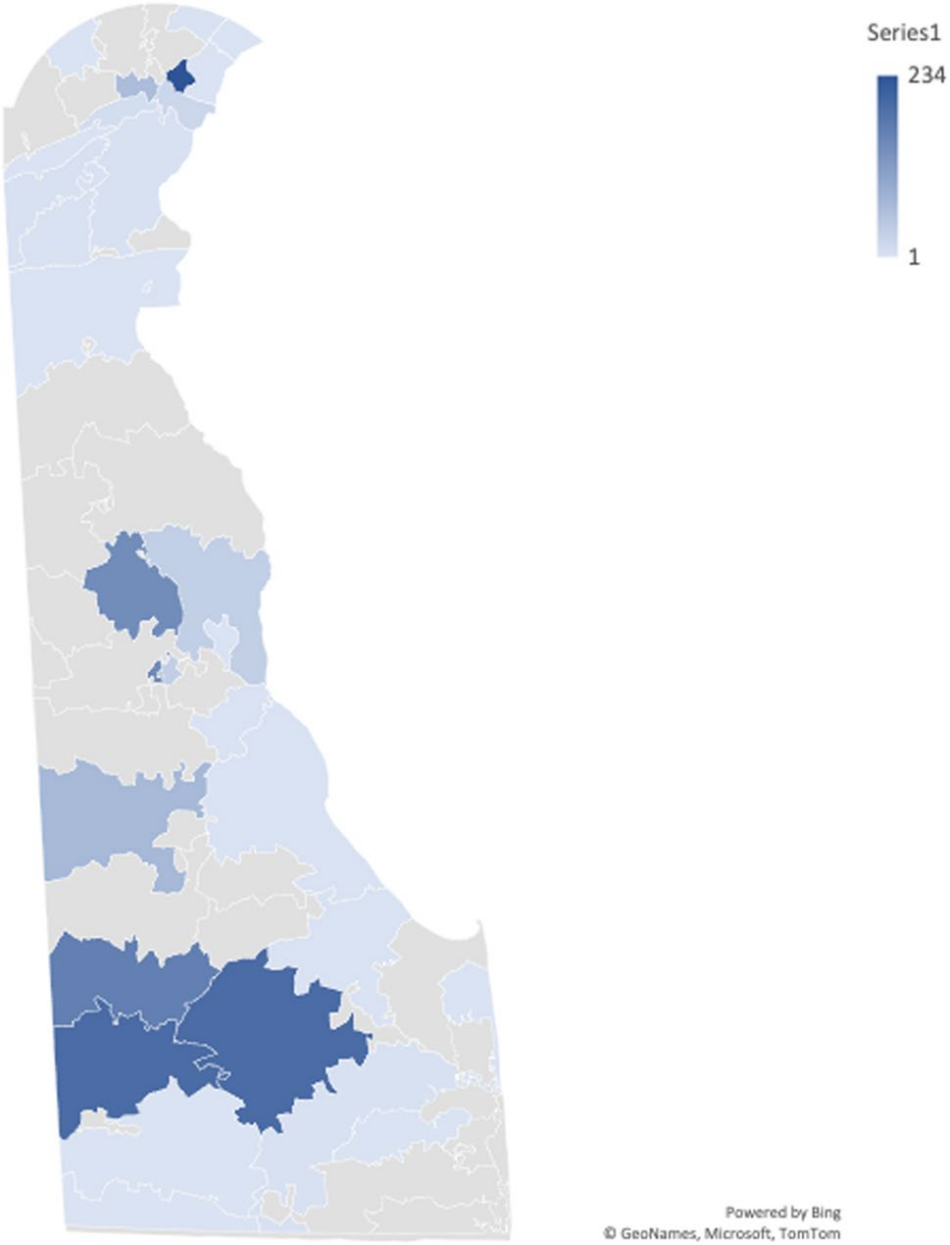
Sample distribution by ZIP code. The map was created based on the ZIP code information provided by 1,109 participants. Fifty-eight participants did not indicate ZIP code, and their location was based on ZIP codes of the study sites

Data were extracted from a retrospective survey conducted between March 4, 2021 and October 30, 2021. All study participants were 18 years of age or older.^1^ The survey contained questions about demographic and socioeconomic characteristics, COVID-related beliefs and practices, general health, COVID-19 testing history, and vaccine-related questions. Questions on the survey were adopted from the COVID-19 Community Response Survey developed by John Hopkins University [14] and NIH Common Data Elements (CDEs). Surveys were conducted electronically through REDcap and using iPads at the study sites.^2^ After excluding participants who reside outside Delaware, the sample size for the study was 1,167. Figure 1 displays a map showing the residency of the sample based on their ZIP codes. It should be noted that those ZIP codes fall within the disadvantaged areas of Delaware that were targeted in our study.

### Measures

**Fig. 2:**
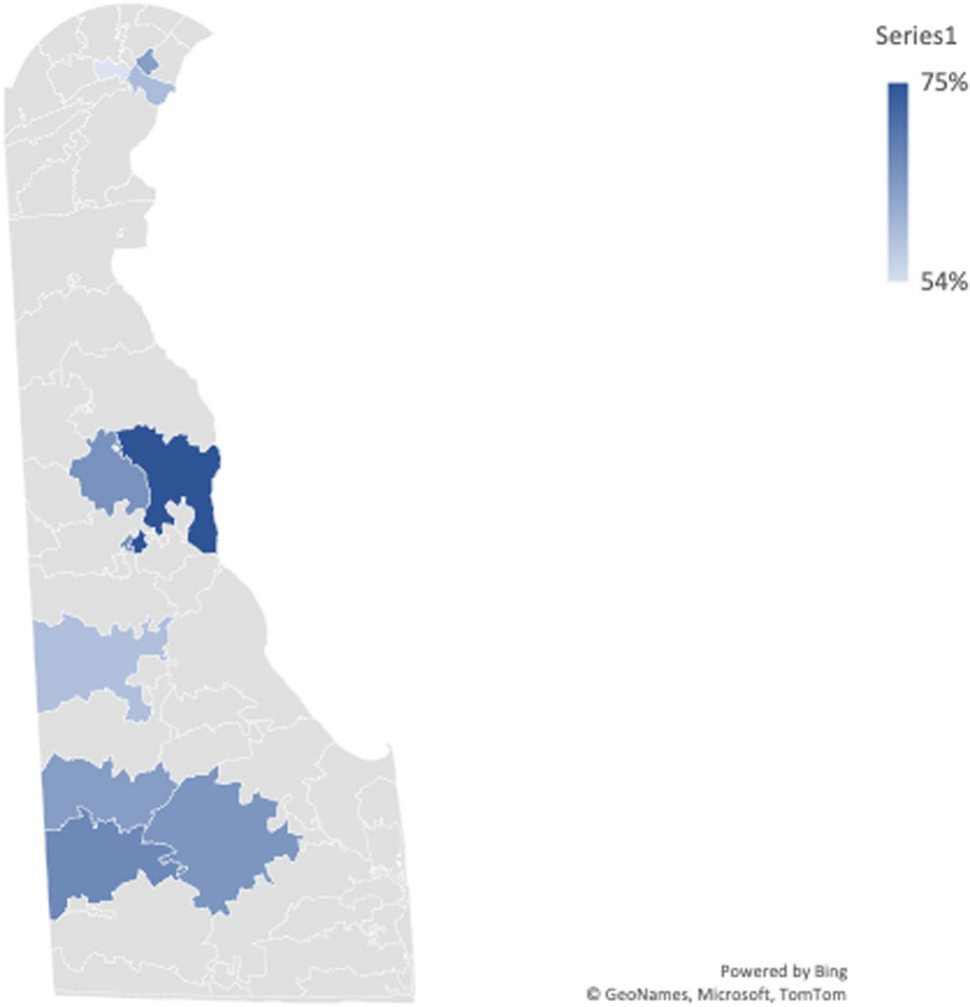
Percentage of sample who have tested for COVID-19 by ZIP code

The outcome variable of interest is a binary variable indicating whether the respondents have been previously tested for COVID. The question on the survey asked: “Have you ever been tested for COVID?” The answer Yes was coded as 1 and No was coded as 0. Figure 2 displays the percentage of respondents by ZIP code who had been previously tested for COVID. It should be noted that some ZIP codes had fewer than 10 respondents. Therefore, to eliminate bias, we only calculated the percentage of COVID testing among ZIP codes with more than 10 respondents. The results indicated that the highest percentage of individuals previously tested for COVID in a given area was 75% (for ZIP code: 19901) and the lowest percentage was 54% (for ZIP code: 19805).

The other outcome variable of interest was the result of the antibody serology test we administered as part of the study (positive = 1, negative = 0). The serology antibody tests were performed to determine past infection. Each participant in the study received a serology test at the study site after completing the survey. Nurses conducted the antibody serology test using lateral flow immunoassays to detect the presence of IgG and IgM antibodies that are typically found in individuals with past COVID infection. However, the antibody test might also show a positive result among those who had recently been vaccinated for COVID-19. Therefore, for the purposes of the present analysis, we coded a serology test as positive if the participant (1) received a positive result on the serology test and (2) had not yet received a single dose of the vaccine.

**Fig. 3:**
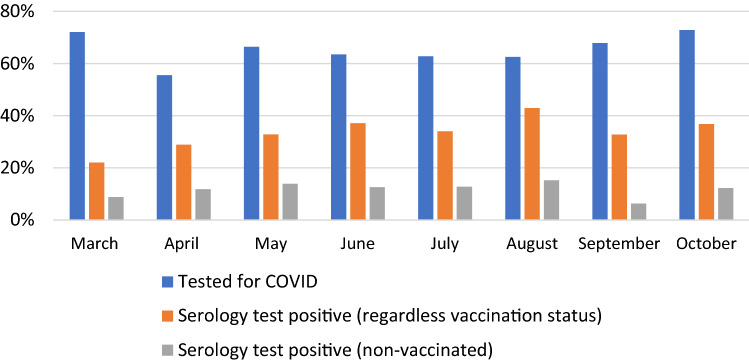
Percentage of participants who received a COVID test in the past and % of participants who received a positive serology test across survey months

Figure 3 summarizes the percentages of participants who had previously been tested for COVID and received a positive result on their serology antibody test at our study site across the survey months (i.e., March 2021–October 2021). The chart indicates a fairly similar pattern of results across the survey months. For informational purposes, the chart also includes two columns: one column indicates the percentage of positive serology test results for all participants (including those who were vaccinated), whereas the other column only shows positive test results for the unvaccinated participants. Notably, in the column that includes vaccinated participants, there is a much higher percentage of positive results than the column that excludes vaccinated participants. This is due to the fact that vaccinated people have similar antibodies in their immune systems as people who had been previously infected with COVID-19. The serology test cannot distinguish between the antibodies resulting from COVID-19 vaccination vs. COVID-19 infection. For this reason, the data analyses described below use serology test data only from the unvaccinated participants.

### Covariates

Several demographic, social, and COVID-related characteristics served as covariates in the analyses. Demographic variables included race and ethnicity (i.e., non-Hispanic White, non-Hispanic Black, Hispanic, and Others/Unknown) and being female (yes = 1). The categories of race and ethnicity are mutually exclusive. The “Other Race/Unknown” category included respondents identifying as Asian (n = 4), Native American (n = 24), Pacific Islander (n = 1), Multi-race (n = 40), other race (n = 4), or unknown (n = 55). The socioeconomic variables included educational level as a categorical variable (i.e., less than high school, high school diploma, some college, and bachelors degree or higher), county of residence as a categorical variable (i.e., New Castle, Kent, or Sussex), family income in 2020^3^ as a categorical variable indicating whether respondents’ family income in 2020 was higher or lower than $20,000 or unknown (unknown = 0, family income $20k and higher = 1, family income lower than $20k = 2), health insurance status as a binary variable (no insurance = 0), and perceived neighborhood safety as a binary variable (yes = 1). It should be noted that family income was included on the survey as a categorical variable. Due to the socioeconomic characteristics of our sample, many respondents might not know their income or have no source of income; therefore, the survey received a relatively large number of “prefer not to answer” or missing values for family income. Considering that no response for family income might serve as a proxy for the respondent’s socioeconomic status, we categorize “prefer not to answer” and missing values for family income to “unknown family income” and use it as a reference category for family income. In addition, neighborhood safety might influence whether the person goes out to get tested. Research has shown that neighborhood safety has a substantial influence on residents’ physical activity [15], and living in an unsafe neighborhood might limit an individual’s ability or willingness to go out and get tested for COVID-19. In our survey, COVID-related items included whether the participant knows where to get tested (yes = 1), whether getting tested was easy (yes = 1), whether the participant received at least one dose of the COVID vaccine (yes = 1), whether the participant is an essential worker (yes = 1), vaccine hesitant (yes = 1), had previously tested positive for COVID (yes = 1), wears a mask (yes = 1), is able to quarantine if they were to test positive (yes = 1), able to isolate if they were to test positive (yes = 1), and their score on the Fear of COVID-19 scale (FCV-19S). It should be noted that the COVID vaccine became available to all Delawareans aged 16 and older on April 6^th^ 2021. Data for this study was collected from March 4, 2021 to October 30, 2021. Considering the timeline of recruitment and vaccine availability, our respondents were eligible to receive COVID-19 vaccines for the majority of the study’s duration. Therefore, most of our data pertaining to vaccine willingness was recorded at a time when COVID-19 vaccines were readily available.

The FCV-19S is a validated mental health questionnaire, measuring an individual’s COVID-related fears [16]. The FCV-19S includes seven items (each being a statement about COVID), and participants are instructed to rate their level of agreement with each statement. The seven items are: (1) I am most afraid of Corona; (2) It makes me uncomfortable to think about Corona; (3) My hands become clammy when I think about Corona; (4) I am afraid of losing my life because of Corona; (5) When I watch news and stories about Corona on social media, I become nervous or anxious; (6) I cannot sleep because I’m worrying about getting Corona; and (7) My heart races or palpitates when I think about getting Corona. The respondent rates their level of agreement with each statement using a 5-point Likert scale, including “strongly disagree,” “disagree,” “neutral,” “agree,” and “strongly agree.” The FCV-19S score ranges from 7 to 35 and is calculated by adding up the responses to each item (i.e., 1–5). A higher score indicates a higher level of fear about COVID-19. We used the recommended cutoff score of 16.5 in our analysis. Previous research has proposed that a score of 16.5 or higher significantly predicts presence of stress/anxiety symptoms [17]. Considering how the pandemic changes over time (e.g., in terms of changing COVID policies and case numbers), we included survey month in the analysis in order to control for potential effects of passage of time.

### Methods

All statistical analyses were performed using STATA. We used logistic regression models to predict the likelihood of having previously tested for COVID and the likelihood of receiving a positive serology test. The logistic regression model allowed us to establish relationships between a binary outcome variable (i.e., having tested for COVID and a positive serology test) and a group of predictor variables (i.e., demographic, socioeconomic, and COVID-related characteristics). In the analysis, ${p}_{test}$ is the probability of having tested for COVID (Yes = 1), while ${\chi }_{11}, {\chi }_{21}$, ${\chi }_{31}$ and ${\chi }_{41}$ are the predictor variables. Specifically, ${\chi }_{11}$ represents the demographic variables, including race/ethnicity and sex; ${\chi }_{21}$ represents the socioeconomic variables, including educational level, family income in 2020, health insurance status, perceived neighborhood safety, and county of residence; ${\chi }_{31}$ represents the COVID-related variables, including having received at least one dose of the COVID vaccine, meeting the cutoff score for FCV-19S, knowing where to get tested for COVID, whether or not it was easy to get tested, being an essential worker, and being able to quarantine or isolate without losing one’s job if they were to test positive for COVID; and ${\chi }_{41}$ represents the survey month variables. Lastly, ${\beta }_{01}$, ${\beta }_{11}$, ${\beta }_{21}$, ${\beta }_{31}$, and ${\beta }_{41}$ are the parameter coefficients estimated through maximum likelihood method in the following equation:

$$logit\left({p}_{test}\right)=\mathrm{log}\left(\frac{{p}_{test}}{1-{p}_{test}}\right)= {\beta }_{01}+{\beta }_{11}{\chi }_{11}+{\beta }_{21}{\chi }_{21}+{\beta }_{31}{\chi }_{31}+{\beta }_{41}{\chi }_{41}$$

For the other outcome of interest (a positive serology test result), ${p}_{postive\_sero}$ is the probability of the serology test outcome being positive (Yes), while ${\chi }_{12}, {\chi }_{22}$, ${\chi }_{32}$ and ${\chi }_{42}$ are the predictor variables. The predictor variables of ${\chi }_{12}, {\chi }_{22}$, ${\chi }_{42}$ are the same as ${\chi }_{11}, {\chi }_{21},$ and ${\chi }_{41}$, respectively. ${\chi }_{32}$ represents the COVID-related variables, including being vaccine hesitant, having previously tested positive for COVID-19, meeting the FCV-19S cutoff score, knowing where to get tested for COVID, whether or not it is easy to get tested, being an essential worker, and being able to quarantine or isolate without losing one’s job if they were to test positive for COVID.${\beta }_{02}$, ${\beta }_{12}$, ${\beta }_{22}$, ${\beta }_{32}$, and ${\beta }_{42}$ are the parameter coefficients estimated through maximum likelihood method in the following equation:

$$logit\left({p}_{postive\_sero}\right)=\mathrm{log}\left(\frac{{p}_{postive\_sero}}{1-{p}_{postive\_sero}}\right)= {\beta }_{02}+{\beta }_{12}{\chi }_{12}+{\beta }_{22}{\chi }_{22}+{\beta }_{32}{\chi }_{32}+{\beta }_{42}{\chi }_{42}$$

## Results

Table 1 displays the descriptive results of the sample. The sample was 53% non-Hispanic Black, 31% non-Hispanic White, 6% Hispanic, and 11% other. In addition, 30% of the respondents were from New Castle County, 21% from Kent County, and 49% from Sussex County. Forty-nine percent of the respondents identified as female. In terms of socioeconomic characteristics, 25% had less than a high school diploma, 38% a high school diploma, and 37% some college education or higher. Slightly less than half of the participants (i.e., 45%) indicated that their annual family income was less than $20,000 in 2020, indicating many of our participants had lower income. About 10% of respondents did not have health insurance, and about 85% of the respondents reported that they felt their neighborhood was safe. Regarding COVID-related characteristics, 42% of our participants had received at least one shot of the COVID vaccine, while 41% were categorized as vaccine hesitant. Importantly, the proportion of vaccine hesitant respondents in our survey might be higher than that observed in other areas. As noted earlier, many of the participants in our study live in underserved neighborhoods with low average income and educational level, and these socioeconomic characteristics might contribute to vaccine hesitancy [18]. In addition, 40% of our participants met the FCV-19S cutoff score, suggesting substantial fear of COVID-19 and higher likelihood of stress/anxiety symptoms. This finding is consistent with a previous study indicating that underserved populations might experience a higher level of mental health issues due to the pandemic [19]. Twenty-three percent of the participants identified as an essential worker. Sixty percent of the participants indicated that they would be able to quarantine without losing their job, and 61% indicated that they would be able to isolate without losing their job. Sixteen percent of the participants did not know where to get a COVID test in their community, and 15% did not believe it was easy to get tested for COVID. Lastly, 84% of the respondents indicated they wore a mask in public.

**Table 1:** Sample characteristics

	Size (%)	Size (#)
Demographic variables		
Race and ethnicity		
Non-Hispanic white	31.25	350
Non-Hispanic black	53.13	620
Hispanic	6.08	71
Others/Unknown	10.80	126
Total	100	1167
County		
New castle	30.08	351
Kent	21.24	249
Sussex	48.59	567
Total	100	1167
Female	48.67	568
Socioeconomic variables		
Educational level		
Less than High school and unknown	25.19	294
High school	37.87	442
Some college and higher	36.93	431
Total	100	1167
Family Income less than $20K in 2020	45.42	530
Family income more than $20K in 2020	33.42	390
Unknown of family income in 2020	12.16	247
Total	100	1167
Feels neighborhood is safe	84.15	982
No insurance	9.68	113
COVID-related variables		
Have received at least one dose of vaccine	41.82	488
Vaccine Hesitant	41.22	481
Met Fear of COVID cutoff score	39.76	464
Essential worker	22.62	264
Able to quarantine without losing job	60.15	702
Able to isolate without losing job	61.35	716
Does not know where to get COVID test	15.95	178
Not easy to get tested for COVID	14.88	166
Wearing a mask in public	83.80	978
Received a COVID test in the past	64.70	755
Positive Serology Test (unvaccinated only)	11.91	139
Sample size	100	1167

Table 2 presents descriptive results by county, including New Castle County, Kent County, and Sussex County. Note that 67% of respondents from New Castle identified themselves as non-Hispanic Black and about 10% identified as Hispanic, whereas 44% of respondents from Sussex self-identified as non-Hispanic Black and 5% as Hispanic. In terms of socioeconomic variables (i.e., education, family income, neighborhood safety, and insurance status), results indicated that respondents from New Castle County have lower socioeconomic status compared to respondents from Kent County and Sussex County. In addition, in terms of COVID-related variables, 27% of respondents from New Castle County have received at least one dose of the COVID-19 vaccine. This number is lower relative to Kent County and Sussex County (i.e., 49.17% and 50.73%). Furthermore, 53% of respondents from New Castle County were classified as vaccine hesitant, higher than Kent County and Sussex County (i.e., 34% and 37%). Lastly, among unvaccinated individuals, 17% from New Castle County had a positive serology test, higher than Kent County (11%) and Sussex County (9%).

**Table 2:** Sample characteristics by County

	New Castle	Kent	Sussex
Race and ethnicity (%)			
Non-Hispanic white	13.68	31.33	39.51
Non-Hispanic black	66.95	54.22	44.09
Hispanic	9.69	2.81	5.29
Others/Unknown	9.69	11.65	11.11
Total	100	100	100
Female (%)	40.76	49.58	56.36
Socioeconomic variables (%)			
Educational level			
Less than high school and unknown	27.92	25.70	23.28
High school	45.30	34.94	34.57
Some college and higher	26.78	39.36	42.15
Total	100	100	100
Family Income less than $20K in 2020	50.43	52.61	39.15
Family income more than $20k in 2020	24.50	30.12	40.39
Unknown of family income in 2020	25.07	17.27	20.46
Feels neighborhood is safe	80.06	81.12	88.01
No insurance	12.82	8.43	8.29
COVID-related variables (%)			
Have received at least one dose of vaccine	27.35	49.17	50.73
Vaccine hesitant	53.28	33.73	37.04
Met Fear of COVID cutoff score	41.88	40.96	37.92
Essential worker	18.23	19.28	26.81
Able to quarantine without losing job	53.56	60.64	64.02
Able to isolate without losing job	54.99	62.25	64.90
Does not know where to get COVID test	17.09	15.26	14.11
Not easy to get tested for COVID	15.95	12.85	13.76
Wearing a mask in public	83.48	83.94	83.95
Received a COVID test in the past	63.53	65.06	65.26
Positive Serology Test (unvaccinated only)	16.52	11.24	9.35
Sample size	351	249	567

Table 3 displays results from the logistic regression models predicting the likelihood of having had a COVID-19 test in Delaware’s underserved communities. Odds ratios are reported. The odds ratio for being non-Hispanic Black was 1.3724 (SE = 0.2258, p = 0.054), and the odds ratio for being Hispanic was 1.7059 (SE = 0.5385, p = 0.091). Results indicated that being non-Hispanic Black and Hispanic increased the likelihood of having been previously tested for COVID in the past when compared to being non-Hispanic White, while holding the covariates constant. The odds ratio for being female was 1.3643 (SE = 0.1898, p = 0.026), suggesting that being female increased the odds of being previously tested for COVID by 36% compared to those who did not identify as female (males and others), while holding the covariates constant. In terms of socioeconomic background, results suggested that compared to lower educated persons (i.e., less than high school), people with at least “some college” education were more likely to get tested for COVID (OR = 1.8800, SE = 0.3503, p = 0.001), while holding other covariates constant. In addition, compared to participants who did not provide their family income on the survey, those with an annual family income lower than $20,000 in 2020 had an increased likelihood of getting a COVID test (OR = 1.9812, SE = 0.3433, p = 0). Not surprisingly, those without health insurance were about 50% less likely to have been previously tested for COVID (OR = 0.5201, SE = 0.1153, p = 0.003). This may be attributed to concerns about out-of-pocket expenses for a COVID test or lack of a primary care physician among those without health insurance. Lastly, those who felt their neighborhood was safe were about 41% more likely to have received a COVID test (OR = 1.4056, SE = 0.269, p = 0.075), when holding the covariates constant. Previous research has suggested that living in an unsafe neighborhood may limit physical activity. Therefore, if participants perceived that their neighborhood was safe, they may be more likely to go out and get tested for COVID if they felt sick. On the other hand, residents living in unsafe neighborhoods may be less likely to go out and get tested.

**Table 3:** Logistic regression analysis estimating person's likelihood of having been tested for COVID-19 in DE's underserved communities

	(1)	(2)	(3)
	Odds Ratio	Robust SE	P level
Demographic characteristics			
Non-Hispanic white	REF		
Non-Hispanic black	**1.3724 + **	0.2258	0.054
Hispanic	**1.7059 + **	0.5385	0.091
Others/Unknown	0.0913	0.2171	0.702
Female	1.3643*	0.1898	0.026
Male and Non-binary	REF		
Socioeconomic characteristics			
New Castle	REF		
Kent	0.7286	0.1477	0.118
Sussex	**0.7404 + **	0.1209	0.066
Less than High school/Unknown	REF		
High School	1.1294	0.1889	0.467
Some College and Higher	1.8800**	0.3503	0.001
Family income less than $20,000	1.9812***	0.3433	0.000
Family income $20,000 and higher	1.2597	0.2509	0.246
Missing information on fam income	REF		
No health insurance	0.5201**	0.1153	0.003
Feels neighborhood is safe	**1.4056 + **	0.269	0.075
COVID-related characteristics			
Received at least one dose of vaccine	2.2557***	0.3258	0.000
Met cutoff for fear of COVID scale	1.3934*	0.1939	0.017
Does not know where to get COVID test	0.7329	0.1698	0.180
Not easy to get tested for COVID	1.0595	0.2601	0.814
Essential worker	1.6050**	0.2923	0.009
If you were to test positive for COVID			
be able to isolate without losing job	1.3504	0.2682	0.130
be able to quarantine without losing job	0.9425	0.1881	0.767
Survey month	Y		
Intercept	**0.4790 + **	0.1904	0.064
Pseudo R2 = 0.1125			
N = 1,167			

***p < = 0.001, **p < = 0.01, *p < = 0.05 + *p* < = 0.1

Regarding COVID-related characteristics, results indicated that respondents who had received at least one does of the COVID-vaccine had higher odds of getting tested for COVID (OR = 2.2557, SE = 0.3258, p = 0). In addition, participants who met the FCV-19S cutoff score for fear of COVID-19 had higher odds of being previously tested for COVID (OR = 1.3934, SE = 0.1939, p = 0.017). Lastly, being an essential worker also increased the likelihood of having been tested for COVID (OR = 1.6050, SE = 0.2923, p = 0.009).

The other main outcome variable examined in our analyses were the participants’ serology test results. The serology tests were run as part of our study to determine whether each participant had been previously infected with COVID. Considering that the serology test detects COVID antibodies, we excluded participants who had already received at least one dose of the COVID vaccine from the analyses. The serology test cannot adequately distinguish antibodies caused by the COVID vaccine vs. COVID infection. Table 4 displays logistic regression results estimating the likelihood of a positive serology test result among the participants in Delaware’s underserved communities. One result worth noting is that being Hispanic increased the odds of having a positive serology test by 2.5 times (OR = 3.4590, SE = 1.304, p = 0.001), compared to being non-Hispanic White, while holding all other covariates constant. College-educated individuals were less likely to have a positive serology test compared to low-educated individuals (i.e., less than high school). The odds ratio for respondents with “some college” education or higher was 0.5414 (SE = 0.1624, p = 0.041), suggesting that individuals with at least some college education were 50% less likely to get a positive serology test compared to those who did not finish high school. Regarding COVID-related characteristics, those who expressed vaccine hesitancy were almost 4 times likely to have a positive serology test than others (OR = 4.8710, SE = 1.107, p = 0.000). Not surprisingly, those who had previously tested positive for COVID in the past were almost 3 times more likely to have a positive serology test (OR = 3.5251, SE = 0.8638, p = 0.000). Lastly, being an essential worker increased one’s odds of having a positive serology test by 62% (OR = 1.6178, SE = 0.3728, p = 0.037).

**Table 4:** Logistic Regression Analysis Estimating Person's Likelihood of a Positive COVID-19 Serology Test in DE's Underserved Communities

	(1)	(2)	(3)
	Odds Ratio	Robust SE	P level
Demographic characteristics			
Non-Hispanic white	REF		
Non-Hispanic black	1.4135	0.3768	0.194
Hispanic	3.4590**	1.3040	0.001
Others/Unknown	1.3153	0.5213	0.489
Female	0.9936	0.2102	0.976
Male and Non-binary	REF		
Socioeconomic characteristics			
New Castle	REF		
Kent	0.8900	0.2500	0.690
Sussex	**0.6627 + **	0.1623	0.091
Less than High school/Unknown	REF		
High School	0.7334	0.1799	0.206
Some College and Higher	0.5414*	0.1624	0.041
Family income less than $20,000	0.8369	0.2227	0.503
Family income $20,000 and higher	0.6406	0.2043	0.163
Missing information on fam income	REF		
No health insurance	1.5911	0.4715	0.117
Feels neighborhood is safe	0.8519	0.2556	0.593
COVID-related characteristics			
Vaccine Hesitant	4.8710***	1.1070	0.000
Positive COVID test in the past	3.5251***	0.8638	0.000
Met cutoff for fear of COVID scale	0.8493	0.1787	0.438
Does not know where to get COVID test	1.6591	0.5671	0.139
Not easy to get tested for COVID	0.9253	0.3236	0.824
Essential worker	1.6178*	0.3728	0.037
* If you were to test positive for COVID*			
be able to isolate without losing job	0.9503	0.3100	0.876
be able to quarantine without losing job	1.7038	0.5566	0.103
Wearing a mask in public	0.7113	0.2190	0.269
Survey month	Y		
Intercept	0.0278***	0.019	0.000
Pseudo R2 = 0.1610			
N = 1,167			

***p < = 0.001, **p < = 0.01, *p < = 0.05 + *p* < = 0.1

## Discussion & conclusion

The COVID-19 pandemic has interrupted people’s lives at the global level, and testing has proven to be an important weapon in combatting this pandemic. Our exploratory research indicates that demographic, socioeconomic, and COVID-related characteristics predict one’s likelihood of having previously tested for COVID or having a past COVID infection. It is worth noting that this study focuses on people residing in underserved communities. However, we found that, even in communities with relatively homogeneous socioeconomic backgrounds, individual-level differences in socioeconomic characteristics may still predict a person’s likelihood of having been tested for COVID and contracting COVID. Our study has significant public health and policy implications.

Regarding self-reported COVID-19 testing history, our results from the logistic regression models indicated that being non-Hispanic Black or Hispanic increased the odds of having been tested previously for COVID, compared to being non-Hispanic White. Nationwide, Black and Hispanic individuals are more likely to contract COVID-19, more likely to be hospitalized due to COVID, and also more likely to die from the disease [9]. Testing and vaccination disparities characterized by race remain high in the U.S. [20, 21]. Focusing on underserved communities, our analysis revealed that Black and Hispanic Delawareans have greater odds of having been tested previously for COVID compared to non-Hispanic White Delawareans. This might be attributed to the fact that Black and Hispanic individuals are more likely to be exposed to the virus and show symptoms due to their occupation or living arrangements. For example, one study found that, among those with Medicaid, Hispanic/Latin individuals had much higher testing rates compared to non-Hispanic White individuals [22].

In addition, our study found that being female, being educated, and feeling safe in their neighborhood increased one’s odds of having been previously tested for COVID-19. These results are consistent with other studies. For example, researchers found that, compared to men, women were more likely to perceive the current pandemic as a serious health disaster and to comply with preventive measures [23]. Therefore, consistent with the idea that women might be on higher alert regarding pandemic-related matters, they might also be more likely to get tested for COVID-19, compared to men. By the same token, being more educated might be correlated with greater knowledge and appreciation of public health concerns as well as greater awareness about the COVID pandemic specifically, making higher educated people more likely to get tested and vaccinated. Not surprisingly, our results revealed that those who had received at least one dose of the COVID-19 vaccine were 2 times more likely to have been tested, compared to those who did not receive the vaccine. People who are willing to get vaccinated for COVID might also display greater confidence in public health measures and preventative strategies, thus making them also more likely to have been tested for COVID. For example, Wang and colleagues found that in Delaware’s underserved communities, those who have been tested for COVID were also more likely to receive COVID vaccines [18]. Lastly, in the current study, we found that essential workers have higher odds of having been tested for COVID than non-essential workers. This might be attributed to the fact that essential workers may interact with more people on a daily basis and are therefore more likely to be exposed to the virus. Some workplaces might also require that essential workers get tested regularly.

Regarding the results of the COVID-19 antibody serology tests, our findings revealed that Hispanic Delawareans were 3 times more likely to have a positive serology test compared to non-Hispanic White Delawareans, indicating the Hispanic respondents were more likely to contract the virus. This result is largely consistent with other recent reports using national datasets. As of November 22, 2021 (before the outbreak of the omicron variant), the CDC reported that Hispanic individuals accounted for 25.1% of all COVID-19 cases in the U.S. (total = 39,145,832). This is a disproportionately high figure compared to the distribution of Hispanic residents in the U.S. general population. Similar disparities have been observed in the state of Delaware. Specifically, Hispanic people accounted for about 25% of positive COVID cases in Delaware.

Moreover, in the present study, educated individuals were less likely to have a positive serology test compared to the less-educated. More highly educated individuals might be more cautious in terms of practicing COVID-19 preventative behaviors. They might also have a higher level of health literacy and greater access to information about COVID. These factors might contribute to a lower likelihood of contracting the virus. Not surprisingly, those who expressed hesitancy about getting vaccinated for COVID-19 were almost 5 times more likely to have a positive serology test and to have contracted the virus. It is also possible that those who were hesitant to get vaccinated took the pandemic less seriously and were less likely to practice preventative measures, such as social distancing and wearing a mask in public. Lastly, being an essential worker increased the odds of having contracted the virus, compared to being a non-essential worker, most likely due to increased interaction with the public.

Our study represents an important preliminary step in investigating the socioeconomic and demographic factors determining a person’s likelihood of having received a COVID test or having been previously infected with COVID using serology tests. Our study may also have limitations, representing open areas for future research. For instance, our analysis did not include other socioeconomic characteristics that could potentially determine COVID-19 infection, such as marital status and household size. In addition, we excluded age from the analysis, because this variable had a ~ 40% missing response rate.

Given ongoing risks associated with being an essential worker, working with the public and in high-risk environments may be of greater risk to people of color and other marginalized groups [24]. This highlights the need to focus on current inequities and steer healthcare toward a focus that supports optimal individual health outcomes [8]. Early identification of risk factors and improving workplace infection control practices related to COVID can provide better health outcomes for those at risk [24]. Often, those who work shift work do not have adequate healthcare or even time to seek healthcare due to work constraints and demands. Therefore, it is most beneficial for organizations and companies with essential workers to provide better educational opportunities and qualified Occupational Healthcare and Safety (OHS) professionals during work hours and over various shifts [25]. While no clear solutions have been specifically identified, the presence of occupational health care workers may be beneficial to the safety, health, and welfare of their employees. OHS professionals can provide employees with free healthcare while at work; be doorkeepers for sick employees via conducting assessments as employees enter the workplace, offer free COVID testing, and implement new policies and procedures to reduce risks for the employees in the workplace. Other benefits include offering free healthcare programs and educational opportunities that are in the best interests of the company and its employees on COVID and other healthcare-related concerns. Implementing various health-related opportunities in the workplace, and fostering the valuable relationships between work and health, may be one solution used to combat existing issues surrounding occupational health and wellness, COVID infections, and associated employee risks.

## Data Availability

The datasets generated during and/or analyzed during the current study are available from the corresponding author on reasonable request.

## Abbreviations

FDA: U.S. Food and Drug Administration
EUA: Emergency Use Authorization
COVID-19: Coronavirus disease 2019
CDC: U.S. Centers for Disease Control and Prevention
CHI: Community Health Index
DE DPH: Delaware’s Division of Public Health
WHC: Wilmington Hope Commission
SCHC: Sussex County Health Coalition
MSN: Masters of Science in Nursing
WHO: World Health Organization
CDEs: NIH Common Data Elements
OHS: Occupational Healthcare and Safety

## Footnotes

1 The ages of respondents in the sample ranged from 18 to 94.

2 Nurses, student interns, staff, and participants wore masks at all times, sanitized their hands and IPADs frequently, and remained 6-feet apart.

3 The question pertaining to family income on the survey read “In 2020, what was your total household income before taxes?”.
